# Progressive Medicine

**Published:** 1904-04

**Authors:** 


PROGRESSIVE MEDICINE, Vol. VI, No. 1, March 1, 1904. Lea Bros. & Co., Philadelphia and New York.

This volume is concerned with the Surgery of the Neck, Head and Thorax, Infectious Diseases, Diseases of Children, Laryngology Rhinology, Otology. The contributors are Chas. H. Frazier, Robert B. Preble, Floyd M. Crandall, Chas. P. Grayson, Robert L. Randolph.

The volume is bound in paper, and is in keeping with the former excellent numbers of the series.

---

### EPILEPSY.

In *Medicine*, for February, W. N. Bullard states that in the treatment of epilepsy bromin in some form seems to be the only generally efficient medicine. Personally he prefers the bromid of sodium, varying it occasionally when necessary with bromid of potassium. The triple bromids are often effective. He gives the medicine before meals, and, if necessary, at bedtime, in a considerable amount of water, a half to one tumblerful at each dose. No unpleasant effects have been observed in ordinary doses, thirty to forty grains a day thus given, except the acne, which is best combatted with Fowler's solution. He believes the most important object must be the regulation of the diet, plenty of exercise, sleep, and occupation of the patient.

